# Preserved MHC Class II Antigen Processing in Monocytes from HIV-Infected Individuals

**DOI:** 10.1371/journal.pone.0009491

**Published:** 2010-03-03

**Authors:** Laila Woc-Colburn, Lavinia Smultea, Lakshmi Ramachandra, David H. Canaday

**Affiliations:** 1 Division of Infectious Diseases and HIV Medicine, Case Western Reserve University School of Medicine, Cleveland, Ohio, United States of America; 2 Department of Pathology, Case Western Reserve University School of Medicine, Cleveland, Ohio, United States of America; 3 Geriatric Research, Education, and Clinical Center, Cleveland Veterans Affairs, Cleveland, Ohio, United States of America; New York University, United States of America

## Abstract

**Background:**

MHC-II restricted CD4+ T cells are dependent on antigen presenting cells (APC) for their activation. APC dysfunction in HIV-infected individuals could accelerate or exacerbate CD4+ T cell dysfunction and may contribute to increased levels of immunodeficiency seen in some patients regardless of their CD4+ T cell numbers. Here we test the hypothesis that APC from HIV-infected individuals have diminished antigen processing and presentation capacity.

**Methodology/Principal Findings:**

Monocytes (MN) were purified by immuno-magnetic bead isolation techniques from HLA-DR1.01+ or DR15.01+ HIV-infected and uninfected individuals. MN were analyzed for surface MHC-II expression and for antigen processing and presentation capacity after overnight incubation with soluble antigen or peptide and HLA-DR matched T cell hybridomas. Surface expression of HLA-DR was 20% reduced (p<0.03) on MN from HIV-infected individuals. In spite of this, there was no significant difference in antigen processing and presentation by MN from 14 HIV-infected donors (8 HLA-DR1.01+ and 6 HLA-DR15.01+) compared to 24 HIV-uninfected HLA-matched subjects.

**Conclusions/Significance:**

We demonstrated that MHC class II antigen processing and presentation is preserved in MN from HIV-infected individuals. This further supports the concept that this aspect of APC function does not further contribute to CD4+ T cell dysfunction in HIV disease.

## Introduction

Activation of CD4+ T cells is dependent on presentation of antigen by MHC-II molecules on APC. Although loss of CD4+ T cells is responsible for the profound T-cell immunodeficiency of AIDS, APC dysfunction in HIV+ individuals could accelerate or exacerbate CD4+ T-cell dysfunction.

Few studies have analyzed the function of APC from HIV-infected and uninfected individuals using antigen-specific T cell responses. *In vitro* infection of MN with very high concentrations of HIV produced significant defects in antigen processing [1], [2]. It is difficult to extrapolate these *in vitro* data to the *in vivo* setting in HIV-infected subjects since the frequency of HIV-infection in macrophages from HIV+ patients was only 1/2,300 to 1/37,000 cells [3], [4]. Two studies from Blauvelt *et al.* measured antigen presentation in HIV-discordant identical twins [5], [6]. In their first paper they studied antigen presentation of influenza by the dendritic cell subtype, Langerhans cells (LC), and MN using T cells from the uninfected twin as the readout of antigen presentation. No defect in the presentation by APC from the three HIV-infected twins was detected. In the second paper the authors found no difference in presentation of tetanus by LC from five HIV-discordant twin pairs. Utilizing a different system, Fidler *et al.* used CD4+ T cell clones from uninfected subjects to measure antigen presentation by MN from HIV-infected individuals. They found a defect in antigen presentation that varied in magnitude depending on which T cell clone they tested [7]. These sets of studies using primary T cells to determine antigen presentation function demonstrate conflicting results. The basis of our study is to clarify the issue of antigen processing function in APC from HIV-infected individuals using a novel and highly sensitive measurement of MHC-II antigen processing and presentation. Additionally, we will determine if there is a relationship between HLA-DR levels and antigen presentation.

HLA-DR restricted CD4+ T cell hybridomas were used to study MHC-II antigen processing. Hybridomas are stable immortal cell lines that provide quantitative and reproducible responses ideal for detailed mechanistic studies [8]. They were generated in HLA-DR transgenic mice, but respond to human APC as previously described [9], and secrete IL-2 in proportion to the amount of specific peptide-MHC on the APC surface. Classically T cell hybridomas are thought to be less costimulation dependent and therefore provide a readout that is largely dependent on levels of expression of peptide:MHC-II complexes only. These murine T cells are also not infectable by HIV. T cell hybridomas respond immediately after thawing from cryopreservation permitting a single large stock of T cells to be used throughout the entire study. MN then become the only variable in the experimental system. We tested the hypothesis that MN from HIV-infected persons are deficient in MHC-II antigen processing and presentation.

## Methods

### Isolation and Storage of PBMC

Recruitment and participation of healthy and HIV-infected subjects was approved by University Hospitals Case Medical Center Institutional Review Board and written informed consent was obtained from all subjects. HIV-infected individuals were recruited from the HIV clinic at the Case Western Reserve Center for AIDS Research. HLA-matched healthy adult donors were identified among personnel at the university and medical center. Blood was collected in EDTA containing tubes and processed within 90 minutes of collection (BD Diagnostics). PBMC were isolated by Ficoll Paque Plus (Amersham Biosciences) density gradient centrifugation and stored in liquid nitrogen in medium containing 90% FCS/10% DMSO. HLA-typing was performed by PCR using kits (Biosynthesis) on DNA isolated from whole blood. Potential subjects first had low resolution screening to identify HLA-DR15 and HLA-DR1 status. Their DNA then underwent high-resolution analysis to confirm that they were HLA-DR15.01 and HLA-DR1.01 subtype. Only subjects that were HLA-DR1.01 and HLA-DR15.01 were studied.

### Isolation of Monocytes

Untouched MN were isolated from thawed aliquots of PBMC using the Monocyte Isolation Kit II (Miltenyi Biotec) according to the manufacture's instruction with the following modifications. Anti-CD1c antibody and anti-CD2 beads were added to minimize contamination by T cells and myeloid dendritic cells. Purity and HLA-DR expression on MN was determined immediately after isolation by flow cytometry after staining with anti-CD14-PE, anti-DR-APC, and isotype control antibodies (BD Biosciences). The average MN purity after isolation was 70% in HIV-infected and 73% in uninfected individuals with no significant difference between the two groups (p>0.5).

### T Cell Hybridoma Generation

T cell hybridomas were generated using IACUC approved protocols with a method that is previously described [8], [9]. Briefly, 15 HEL T cell hybridoma line was generated by immunizing HLA-DR15.03 transgenic mice (Chella David, Mayo Clinic) with Hen Egg Lysozyme (HEL, Roche) in complete Freund's adjuvant (Gibco). After 7 days, draining lymph nodes were harvested and restimulated *in vitro* for 5 days. Cells were fused with a TCR deficient line BW1100 [10] with polyethylene glycol (Boehringer Mannheim). HEL-specific cells were identified by screening for presentation by a HLA-DR15.01+ EBV transformed B cell line. The specific HEL-epitope recognized by 15HEL was determined by testing peptides (Genemed Synthesis, S. San Francisco, CA) that were predicted by to bind to HLA-DR15.01 by the prediction algorithms from ProPed (http://www.imtech.res.in/raghava/propred/) and RANKPEP (http://immunax.dfci.harvard.edu/Tools/rankpep.html). 1ACD5 T cell hybridoma is an HLA-DR1.01 restricted clone that recognizes HIV reverse transcriptase (RT, aa 350–364) as previously described [8], [9].

### T Cell Assay

For the antigen presentation assays, MN (5×10^4^/well) and T cell hybridoma (1×10^5^/well) were plated in 96-well flat bottom plates in triplicate with recombinant RT, HEL, or peptides in DMEM based medium with 10% FCS [11]. After 24h, supernatants were harvested and IL-2 levels measured by ELISA (eBioscience).

## Results

### MHC-II Levels on MN from HIV-Infected Individuals

**Figure 1:**
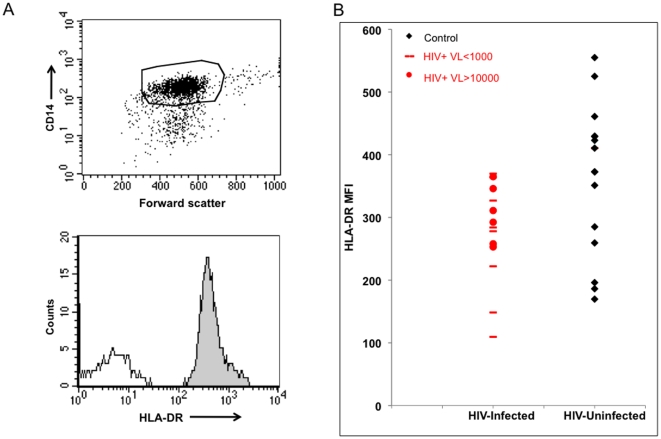
HLA-DR levels on MN from HIV-infected and uninfected individuals. Purified MN were labeled with anti-CD14 to identify the MN and MFI of HLA-DR. A (upper panel). Representative dot plot of the gating strategy of the single CD14+ population is presented. A (lower panel). Representative histogram of HLA-DR (grey) and isotype control (dotted black) levels. B. HLA-DR levels of control and HIV infected subjects. Black circles for viremic individuals (VL>1000), black dash subjects with VL<1000, and red circles uninfected controls.

MHC-II HLA-DR is one of the predominant antigen presentation molecules for CD4+ T cells. HLA-DR levels were measured on purified MN for all subjects by flow cytometry. Fig. 1A demonstrates the gating strategy used to measure HLA-DR on purified CD14+ MN. Mean florescent intensity (MFI) of HLA-DR was found to be significantly less (Fig. 1B, p<0.03) in HIV-infected individuals (MFI 281, SD 79) than uninfected subjects (MFI 352, SD 105). While there was a 20.2% reduction in HLA-DR on MN from HIV-infected group as a whole, HLA-DR levels on MN from viremic subjects (VL>1000) were more similar to those of the control population. We sought to determine the functional consequences of this HLA-DR reduction by measuring antigen presentation in these same MN.

### Characterization of the T Cell Hybridoma Lines

HLA-DR restricted T cell hybridomas were used to measure antigen processing and presentation by MN. T cell hybridomas for use with human APC were characterized in three ways. First was to confirm that the T cell hybridoma recognizes the appropriate protein by mapping the exact peptide recognized. The second was to confirm HLA-DR restriction. Last, the costimulation dependence of the T cell hybridomas was determined.

**Figure 2:**
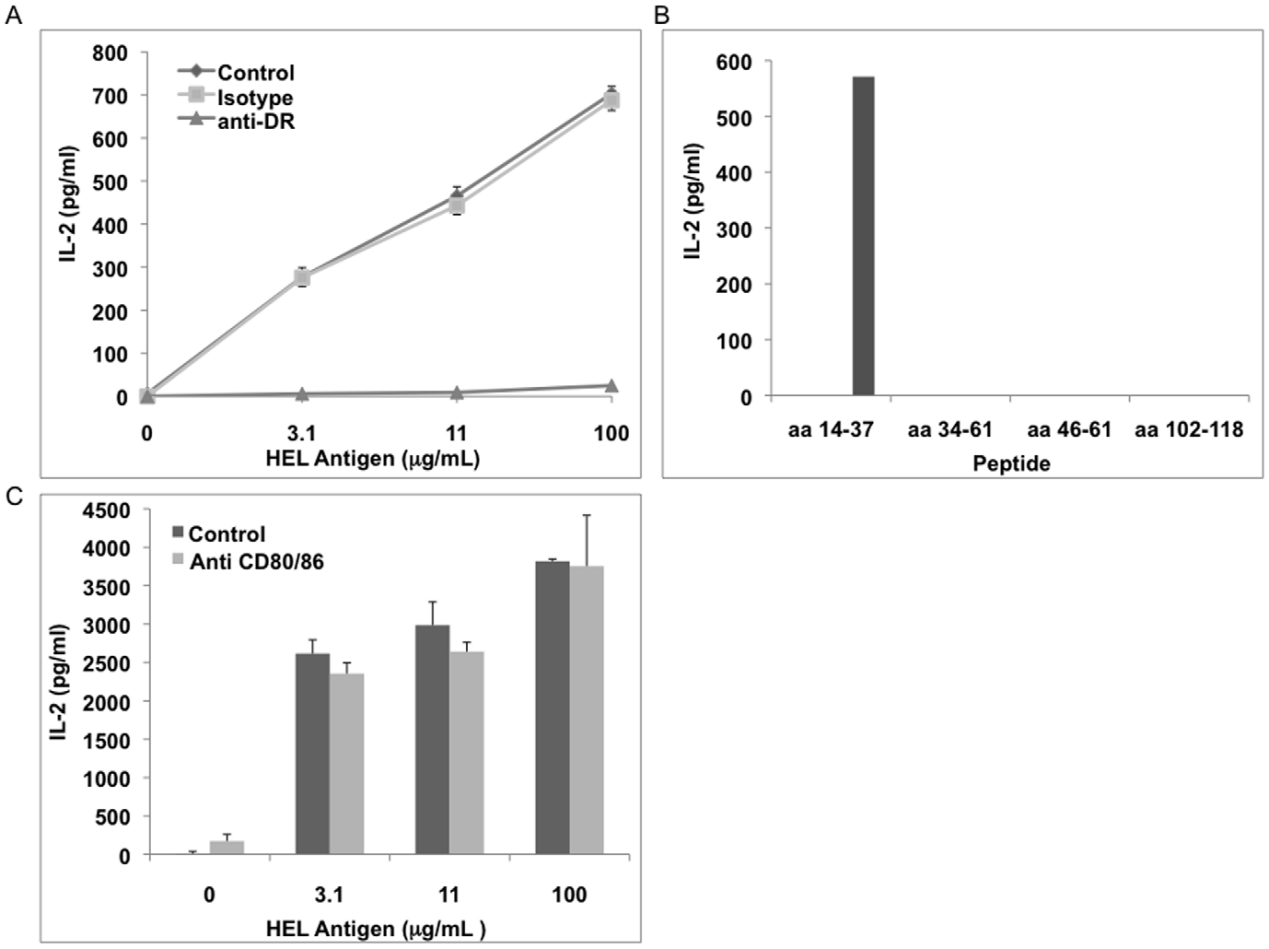
Characterization of T cell hybridoma clone 15HEL. MN (5×10^4^/well) were incubated with antigen (intact HEL for A and C, HEL peptides for B) and 15HEL (1×10^5^/well) for 24 hrs. Supernatants were harvested, and IL-2 measured by ELISA. A. Effect of anti-HLA-DR or isotype on response. B. Identification of HEL peptide epitope recognized by 15HEL. C. Effect of anti-CD80 and CD86 antibodies together (5 µg/ml of each) or isotype control (10 µg/ml) on responses. Results of A and C represent triplicate wells and error bars SEM.

As published previously, the RT-specific T cell hybridoma 1ACD5 recognized RT aa 350–364, was restricted to HLA-DR1.01, and did not require CD80/86 costimulation [11]. For the current study, the peptide specificity of the 15HEL clone was mapped. Rather than purchase an entire set of overlapping peptides, MHC-II peptide binding algorithms were used to predict candidate peptides from HEL that would bind HLA-DR15.01. This limited set of peptides was synthesized and tested. 15HEL responded only to HEL aa 14–37 (Fig. 2A). HLA-DR restriction was confirmed using anti-DR antibodies that significantly inhibited 15HEL responses (Fig. 2B). 15HEL did not recognize non-HLA-DR15 expressing APC (data not shown). The addition of anti-CD80/86 antibodies only minimally diminished IL-2 production by 15HEL demonstrating that 15HEL activation by MN was not CD80/86 costimulation dependent (Fig. 2C).

### Antigen Processing and Presentation by MN from HIV-Infected Individuals

The goal of these studies was to determine if MN from HIV-infected individuals have an antigen processing and presentation defect. Processing and presentation by the HLA-DR1.01+ and DR15.01+ MN were analyzed separately since two different T hybridomas were used to measure antigen presentation. Table 1 describes the clinical features of 8 HLA-DR1.01+ and 6 HLA-DR15.01+ HIV-infected subjects studied. 12 HLA-DR1.01+ and 12 HLA-DR15.01+ uninfected healthy controls served as the control group for comparison.

**Table 1:** 

SUBJECT	VLOAD	CD4+	ARV
HIV1	<50	785	Yes
HIV2	57	1476	Yes
HIV3	>100000	56	No
HIV4	<50	772	Yes
HIV5	4810	685	Yes
HIV6	<50	576	Yes
HIV7	<50	1342	Yes
HIV8	21300	228	No
HIV10	<50	104	Yes
HIV11	10600	28	No
HIV12	794	218	Yes
HIV13	8240	221	No
HIV14	<50	185	Yes
HIV16	99400	426	No

**Figure 3:**
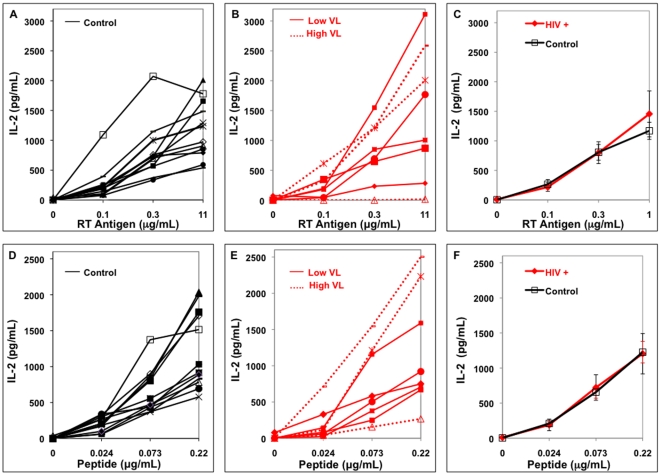
Antigen presentation by HLA-DR1+ MN from individual HIV-infected and uninfected subjects. In all four panels, MN (5×10^4^/well) were incubated with soluble antigen or peptide and T cell hybridoma (1×10^5^/well) for 24 hrs. Viremic HIV-infected individuals with VL>1000 are High VL and VL<1000 are Low VL. A. Control MN and soluble RT, B. HIV+ MN and soluble RT, C. Combined data on soluble RT presentation, D. Control MN and peptide, E. HIV+ MN and peptide, F. Combined data on peptide presentation.

**Figure 4:**
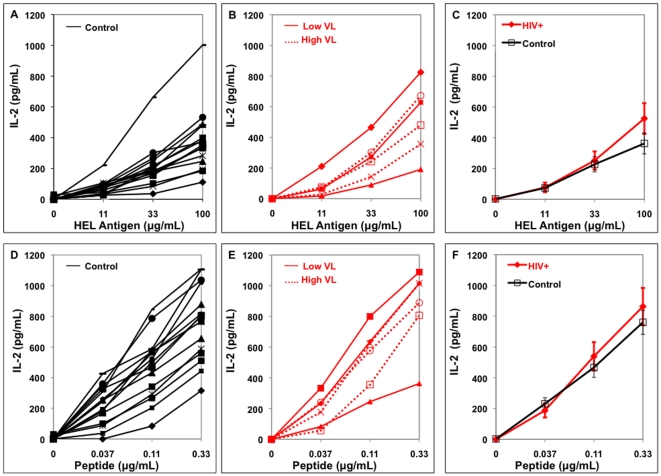
Antigen presentation by HLA-DR15+ MN from individual HIV-infected and uninfected subjects. MN (5×10^4^/well) were incubated with soluble HEL or peptide and T cell hybridoma (1×10^5^/well) for 24 hrs. Viremic HIV-infected individuals with VL>1000 are High VL and VL<1000 are Low VL. A. Control MN and soluble HEL, B. HIV+ MN and soluble HEL, C. Combined data on soluble HEL presentation, D. Control MN and peptide, E. HIV+ MN and peptide, F. Combined data on peptide presentation.

Antigen presentation assays were performed using several concentrations of soluble antigen and peptide. Soluble antigen must be taken up, processed, and presented while peptide can be exchanged on the MN surface and does not require intracellular processing to be presented. Fig. 3 and 4 demonstrate presentation of soluble antigen and peptide by MN from HIV-infected and uninfected control donors of HLA-DR1 (Fig. 3) and HLA-DR15 types (Fig. 4). Responses by the uninfected-controls and HIV-infected individuals for each HLA-DR type were compared as a group (Fig. 3C, 3F, 4C, 4F). There was no statistical difference between antigen presentation from HIV-infected and uninfected groups at every soluble antigen or peptide concentration tested (p>0.1).

The study did not have the power to examine the effects specifically of viremia or use of ARV on antigen presentation. The individual viremic subjects were indicated on the figure of raw data of all the subjects (Fig. 3 and 4). One viremic donor (HIV 5) on self-report said that he was taking ARV while all the other viremic individuals did not report current ARV use. Based on the presentation by the individual subjects in Fig. 3 and 4, there does not appear to be a clear trend that viremia or use of ARV results in loss of antigen presentation ability compared to uninfected controls. In addition, there was no relationship between antigen presentation and CD4+ T cell level (data not shown).

**Figure 5:**
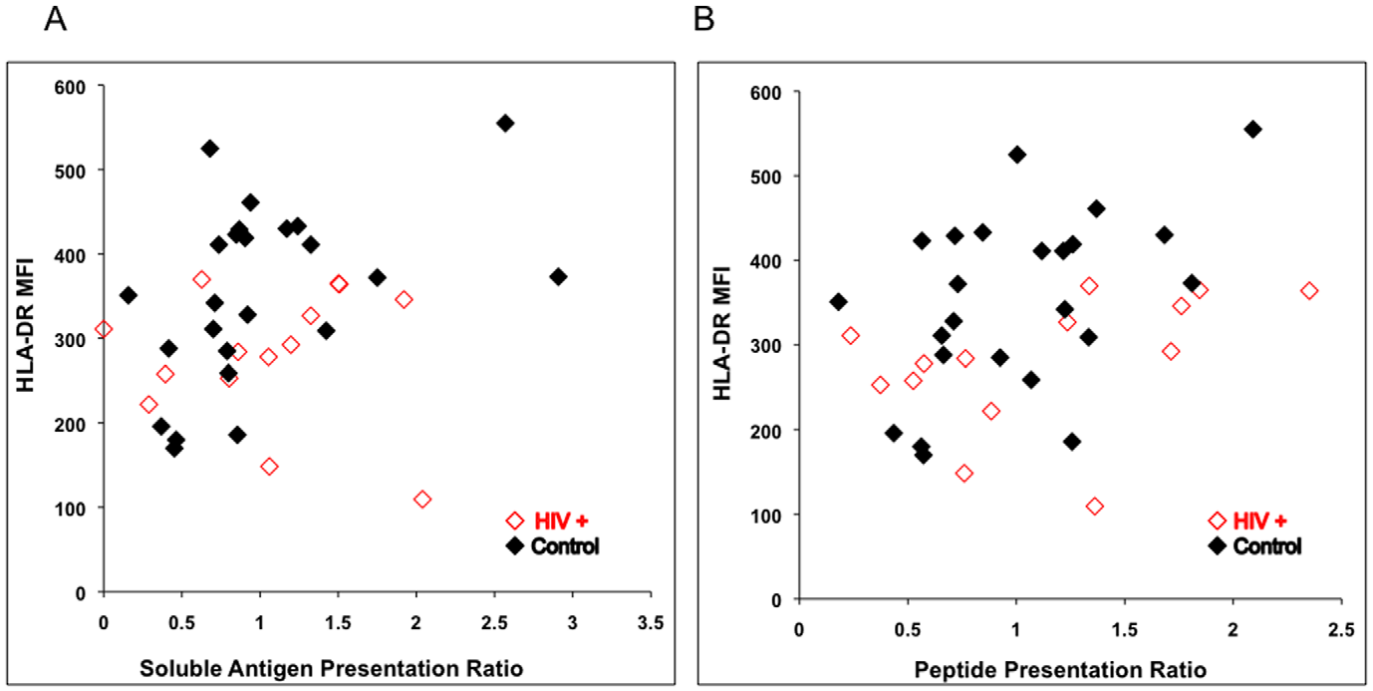
Relationship between HLA-DR level and antigen presentation. Data from all 38 subjects in the study were normalized. The individual's ratio of presentation was calculated by dividing their IL-2 level for the middle concentration of RT, HEL or peptide by the mean IL-2 level from the control subjects at the same middle concentration of antigen or peptide. Open red diamonds are data from HIV-infected individuals and closed black diamonds for uninfected controls.

Since HLA-DR levels were measured on MN for all subjects by flow cytometry, the relationship between HLA-DR level and antigen presentation was determined. In order to increase the power for the analysis, all data from the HLA-DR1 and DR15+ subjects were included (n = 38). Data were normalized to allow this by dividing the mean level of IL-2 produced by the control group in their respective HLA-DR types by the IL-2 value for that same antigen or peptide in each individual subject. Analysis of the middle concentration of antigen and peptide was chosen to determine this correlation because virtually all of the subjects had IL-2 values at this concentration of antigen or peptide that were above the limit of detection of the IL-2 ELISA assay but also not at plateau of the response. The ratio of presentation was then analyzed using Spearman's rank correlation. There was a small but significant correlation of antigen presentation of the soluble antigens and HLA-DR (Fig. 5A, r = 0.32, p = 0.049) and peptide presentation and HLA-DR (Fig. 5B, r = 0.38, p = 0.02). Interestingly, the 20.2% reduction in HLA-DR expression in the HIV-infected individuals (Fig. 1) was not reflected in a drop in antigen presentation however (Fig. 3 and 4).

## Discussion

APC dysfunction could be a significant factor in immune dysfunction since both cell mediated and humoral immunity rely on APC function to activate their specific responses. There have been several *in vitro* studies where macrophages are infected with HIV or transfected to express specific HIV molecules such as Nef and Vpu [2], [12], [13], [14]. These studies have demonstrated multiple inhibitory effects on APC function by HIV virions or specific HIV products. These effects include inhibition of MHC-II expression, increase in invariant chain expression, decrease in antigen uptake, and decreased antigen presentation overall. These studies though all have a similar feature in their experimental systems of very high levels of HIV-infection or expression of a transfected HIV molecule. This is not the case *in vivo* where the frequency of HIV-infected MN and macrophages is very low. In addition it is well known that MN are resistant to becoming productively infected due to a pre-integration block [15]. Both of these features suggest that APC dysfunction if present would be due to bystander effects rather than direct infection. We did not detect a defect in either antigen processing or presentation by MN isolated from HIV+ individuals. Our results using a very specific measurement tool of MHC-II antigen processing and presentation are similar to the observations reported by Blauvelt *et al.* [5], [6].

Several older studies have examined antigen presentation function by APC from HIV-infected individuals using primary human CD4+ T cells. These data must be interpreted with caution because of several confounding factors. HIV infection of the human CD4+ T cell lines is a major potential confounder. Release of modulatory cytokines by APC from HIV-infected individuals affecting T cell responses is also possible. We attempt to minimize these uncontrolled effects through our HLA-DR transgenic murine T cell hybridoma system. These murine T cell lines cannot be infected by HIV and some cytokine or chemokine effects if present might not signal across the xeno barrier. T cell hybridomas are relatively costimulation independent permitting specific measurement of specific peptide-loaded MHC-complexes. All of these features make use of DR-restricted T cell hybridomas an excellent means to measure the generation and presentation of peptide:MHC complexes in isolation from effects on human CD4+ T cells.

Our study demonstrates that antigen processing and presentation of soluble antigen is preserved in MN from HIV-infected individuals. It is difficult to screen and match the HLA-DR type and subtype to have a large number of subjects. It is possible that there are small antigen presentation defects in specific subgroups of individuals that would be detectable with a larger sample size. The current dataset did not establish a relationship between HIV viral load, CD4 count, and antigen presentation.

In the literature there are varying reports from an increased level of HLA-DR on APC from HIV-infected individuals to somewhat diminished levels [5], [16], [17], [18], [19], [20], [21]. It is not clear why MN from HIV-infected individuals have 20% lower HLA-DR but their antigen presentation is not similarly diminished in our study. It is possible that the magnitude of HLA-DR reduction is not great enough to have a measurable effect on antigen presentation with our sample size. However, in previous data examining antigen presentation by cord blood MN [11], we did not find a correlation between antigen presentation and HLA-DR levels. In the current study we found only a small correlation between HLA-DR level and antigen presentation. These data together suggest that intracellular events are the most important determinants of successful antigen processing and presentation and not merely surface MHC-II levels.

In conclusion, our data using the antigen presentation focused T cell hybridoma bioassay system suggests that MN from HIV-infected individuals have preserved MHC-II antigen processing and presentation function.
